# Discrepancies and Quality of Medication Allergy Documentation Between Primary Care and Hospital Information Systems: A Retrospective Cohort Study

**DOI:** 10.3390/ph19081259

**Published:** 2026-08-10

**Authors:** Mohamad Bakor, Elena Ramírez

**Affiliations:** Clinical Pharmacology Department, Faculty of Medicine, La Paz University Hospital-IdiPAZ, Universidad Autónoma de Madrid, 28046 Madrid, Spain; mohamad.bakor@estudiante.uam.es

**Keywords:** patient safety, medication errors, drug allergy, electronic health records, interoperability, continuity of care

## Abstract

**Background and Objective:** Accurate medication allergy documentation is essential for patient safety. This study evaluated discrepancies and documentation quality between medication allergy records in the primary care information system (AP-Madrid) and the Hospital Clinical Information System (HCIS) at a tertiary care hospital. **Methods:** A retrospective cohort study included 223 patients with medication allergy records during 2022. Agreement between systems was assessed using Cohen’s kappa, and documentation quality was classified as adequate, moderate, or poor based on four predefined parameters. **Results:** Agreement between AP-Madrid and HCIS was poor (Cohen’s κ = −0.25). Of the 223 patients, medication allergy records were documented exclusively in HCIS for 94 patients, exclusively in AP-Madrid for 36 patients, and in both systems for 88 patients, while 5 patients had medication allergy information identified only in free-text clinical documentation. Documentation quality was predominantly poor in both HCIS (60.4%, *n* = 134) and AP-Madrid (61.9%, *n* = 138). Beta-lactam antibiotics and non-steroidal anti-inflammatory drugs (NSAIDs) were the most frequently implicated therapeutic groups. Two positive medication re-exposure events were identified. **Conclusions:** Substantial discrepancies and poor documentation quality were identified between primary care and hospital medication allergy records, highlighting limited interoperability between the two systems. Improving the completeness, consistency, and standardized exchange of medication allergy information across healthcare settings may enhance patient safety and continuity of care.

## 1. Introduction

Patient safety is a cornerstone of modern healthcare and is defined as the reduction of unnecessary harm to an acceptable minimum [1]. Despite substantial global efforts to improve healthcare quality, preventable adverse events remain a major public health challenge. It is estimated that approximately three million deaths occur annually due to unsafe healthcare, and nearly one in ten patients experiences harm during the provision of care [2]. Medication-related incidents account for 10–40% of all reported adverse events worldwide, with more than one-third considered preventable [3]. In response, the World Health Organization (WHO) launched the Global Patient Safety Action Plan 2021–2030, which identifies patient safety as a fundamental pillar for reducing avoidable harm and medication-related morbidity and mortality [4].

Medication errors are defined as any preventable event that may cause or lead to inappropriate medication use or patient harm while the medication is under the control of a healthcare professional or consumer. Beyond compromising clinical outcomes, these errors diminish therapeutic effectiveness and impose a substantial economic burden, costing the global healthcare system an estimated USD 42 billion annually [5]. Consequently, the Joint Commission has identified the reduction of medication errors as a National Patient Safety Goal, and the WHO’s third Global Patient Safety Challenge, Medication Without Harm, aims to halve medication-related harm within five years. In addition to medication errors, adverse drug reactions (ADRs) affect up to 10% of the global population and approximately 20% of hospitalized patients [6]. Among these reactions, drug hypersensitivity reactions represent an important clinical concern and account for more than 10% of all ADRs [6]. Among the various adverse drug reactions, drug allergies are one of the most clinically significant. A drug allergy is an atypical hypersensitivity reaction resulting from an exaggerated immune response to a medication [7]. These reactions contribute significantly to patient mortality, accounting for up to 20% of anaphylaxis-related deaths [6,8,9]. While the burden of allergic diseases is rising, the World Allergy Organization notes that over 10% of all adverse drug reactions are unpredictable drug hypersensitivity events. Although a patient’s initial allergic reaction may be unavoidable, subsequent reactions to the same drug or class are predictable and, therefore, preventable [7,10].

From a clinical pharmacology perspective, drug hypersensitivity reactions comprise a heterogeneous group of adverse events encompassing all four types of the Gell and Coombs classification: Type I (IgE-mediated), exemplified by penicillin-induced anaphylaxis; Type II (cytotoxic), such as methyldopa-induced hemolytic anemia; Type III (immune complex-mediated), involving sulfonamides; and Type IV (T-cell-mediated) delayed hypersensitivity reactions, including Stevens–Johnson syndrome associated with allopurinol, carbamazepine, or lamotrigine [9,10]. Despite this complexity, accurate medication allergy documentation remains a critical safeguard for informed clinical decision-making. However, while electronic health records have been implemented to improve documentation and record-keeping [11], healthcare providers frequently document these events inaccurately or incompletely. This often results in the misclassification of predictable pharmacological effects or drug intolerances as true immune-mediated allergies. Such documentation errors lead to the unnecessary avoidance of first-line therapies, particularly antibiotics, forcing the use of less effective alternatives and ultimately driving up healthcare costs and compromising patient safety [12]. Furthermore, computerized clinical decision support systems designed to screen for drug allergies face significant challenges, including inaccurate electronic health record documentation and high-alert override rates driven by low alert specificity [13]. In this context, the recently published 2026 European Society of Clinical Microbiology and Infectious Diseases (ESCMID) clinical guidelines on the evaluation and management of reported antibiotic allergy emphasize the importance of accurate characterization and structured assessment of antibiotic allergy labels. The guidelines note that a substantial proportion of reported antibiotic allergy labels are not supported by evidence of true immune-mediated hypersensitivity. They therefore advocate a structured evaluation of reported reactions, incorporating clinical history and risk assessment to determine the need for further diagnostic testing and, where appropriate, removal of inaccurate allergy labels. These recommendations further reinforce the importance of complete, accurate, and clinically meaningful allergy documentation within electronic health records to support appropriate prescribing, patient safety, and antimicrobial stewardship [14].

In complex hospital settings like La Paz University Hospital, where data from various levels of care converge, discrepancies between primary care records (AP-Madrid) and the Hospital Clinical Information System (HCIS) create significant risks for preventable medication errors. Despite the digitalization of medical records, inconsistencies persist, requiring further investigation into their magnitude and clinical consequences. Evaluating documentation quality is essential as it provides critical clinical information, including reaction severity and symptom duration, required for safe prescribing.

Despite the extensive literature describing deficiencies in drug allergy documentation, previous studies have primarily evaluated medication allergy documentation within a single healthcare setting or compared electronic health records with patient self-reported allergy information rather than assessing discrepancies between independent healthcare information systems. For example, Kabakov et al. evaluated discordance between patient interviews and hospital electronic records in an acute care setting, while Zhou et al. focused on the prevalence and characteristics of allergy documentation within a single healthcare system [15,16]. Similarly, Fernando et al. explored clinicians’ perspectives on allergy documentation practices, whereas Luri et al. conducted a systematic review of drug allergy alert systems, highlighting substantial variability in system structure, terminology, and functionality across electronic health record platforms without evaluating discrepancies between primary care and hospital information systems [12,17]. In addition, Rukasin et al. examined the impact of electronic health record transitions on allergy labels rather than the consistency of medication allergy documentation between different levels of care [18]. In contrast, the present study focuses on discrepancies in medication allergy documentation between primary care and hospital information systems. In both settings, allergy information is routinely documented by healthcare professionals; however, these systems are not fully interoperable, and allergy records are not automatically synchronized. Consequently, updates or modifications made in one system may not be consistently reflected in the other, leading to clinically relevant inconsistencies. This issue is particularly important in integrated healthcare systems such as the Community of Madrid, where patients frequently transition between care settings, making consistent allergy documentation essential for patient safety.

Therefore, this study aims to provide evidence highlighting the need for improving the quality of drug allergy documentation and optimizing interoperability between clinical information systems at La Paz University Hospital, rather than determining the superiority of one system over the other. To address this, the primary endpoint was the level of agreement between primary care (AP-Madrid) and hospital (HCIS) records regarding the presence of medication allergy documentation, measured using Cohen’s kappa coefficient. The secondary endpoint was the assessment of the quality of medication allergy documentation in both systems, classified as adequate, moderate, or poor based on predefined parameters. Additionally, a descriptive analysis of the most frequently implicated drugs and therapeutic groups was performed.

## 2. Results

### 2.1. Allergy Documentation in the Hospital Clinical Information System (HCIS)

Documentation quality was evaluated independently in each information system; therefore, percentages correspond to the distribution within each system.

This study analyzed 223 patients with a mean age of 64.66 years, predominantly female (57.4%). Although a personal history of allergy was reported by 20.6% of patients, a significant data gap was identified: allergy severity remained unknown in over 90% of both clinical evaluations and formal electronic records.

Despite this lack of detail, documentation of recommendations and contraindications was nearly universal (97.3%), with most records originating from the Allergy Department. In terms of therapeutic classes, antibiotics were the most frequently evaluated drug group (50.2%), with beta-lactam penicillins accounting for 22% (*n* = 46), followed by analgesics/pyrazolones (17.7%) (Table 1). The severity documented in the HCIS system was unknown in 91% of cases, whereas mild and severe reactions were documented in 1.3% and 7.6% of cases, respectively.

In the re-exposure analysis, 98.2% of patients had no subsequent exposure to the implicated drug. Only four cases experienced re-exposure, including two positive and two negative cases. However, among the few documented re-exposure events, one case of severe clinical significance was identified: a patient experienced anaphylactic shock following the accidental administration of amoxicillin. This positive re-exposure event was a direct consequence of a critical deficiency in the information system: the patient’s allergy was not recorded in the electronic health record. This omission prevented relevant safety alerts from being triggered during the prescription and administration of the drug. This finding highlights that despite the low frequency of re-exposures (1.8%), documentation failures can transform a known risk into a potentially fatal iatrogenic event.

Notably, the overall quality of the records was classified as poor in 60.4% (*n* = 134) of cases, with only 7.7% (*n* = 17) meeting the criteria for adequate documentation (Table 1).

A total of 209 drug records were registered in the Hospital Clinical Information System (HCIS), as detailed in Supplementary Table S1. According to this updated pharmacological distribution, the most frequently implicated active ingredient at ATC level 5 was metamizole, accounting for 16.27% of the records (34 counts). Within the therapeutic categories, beta-lactam antibiotics constituted a prominent group, led by penicillin G, amoxicillin, and benzylpenicillin, each representing 5.26% of the records (11 counts each), followed by the combination amoxicillin–clavulanate at 3.83% (8 counts). Other frequently recorded anti-infective agents included the cephalosporins ceftriaxone (3.35%, 7 counts) and cefepime (2.87%, 6 counts), as well as the glycopeptide vancomycin (3.35%, 7 counts).

### 2.2. Allergy Documentation in Primary Care (AP-Madrid)

Regarding AP-Madrid, while the study population remains the same as previously described, significant discrepancies were found in allergy documentation. A personal history of allergies was identified in 49.3% of records (*n* = 110). However, similar to the hospital system, clinical characterization was incomplete: allergy severity was unknown in 90.6% of cases, with only 7.6% documented as severe. Recommendations and contraindications were present in 65.5% of the records, leaving nearly one-third of patients (34.5%) without this critical safety information. The primary medication groups undergoing allergy evaluation were antibiotics (36.44%), of which beta-lactam antibacterials (penicillins) represented 20.4% (n=46). This group was followed by NSAIDs (20.44%) and analgesics (15.56%). Medication re-exposure was identified in only one case, which yielded a positive result. Consistent with these findings, 61.9% (*n* = 138) of the records were classified as having poor documentation quality, whereas only 4.0% (*n* = 9) met the criteria for adequate documentation (Table 2).

A total of 225 drug records were registered in the primary care information system (AP-Madrid), as detailed in Supplementary Table S2. According to this pharmacological distribution, the most frequently implicated active ingredient at ATC level 5 was amoxicillin, accounting for 15.56% of the records (35 counts), closely followed by metamizole at 13.78% (31 counts). Within the therapeutic categories, non-steroidal anti-inflammatory drugs (NSAIDs) were highly prevalent, represented primarily by ibuprofen (8.00%, 18 counts), acetylsalicylic acid (6.22%, 14 counts), and naproxen (3.56%, 8 counts). Other frequently recorded agents of clinical relevance included the opioid tramadol (5.33%, 12 counts) and ampicillin (4.44%, 10 counts).

### 2.3. System Interoperability and Overall Agreement

**Figure 1:**
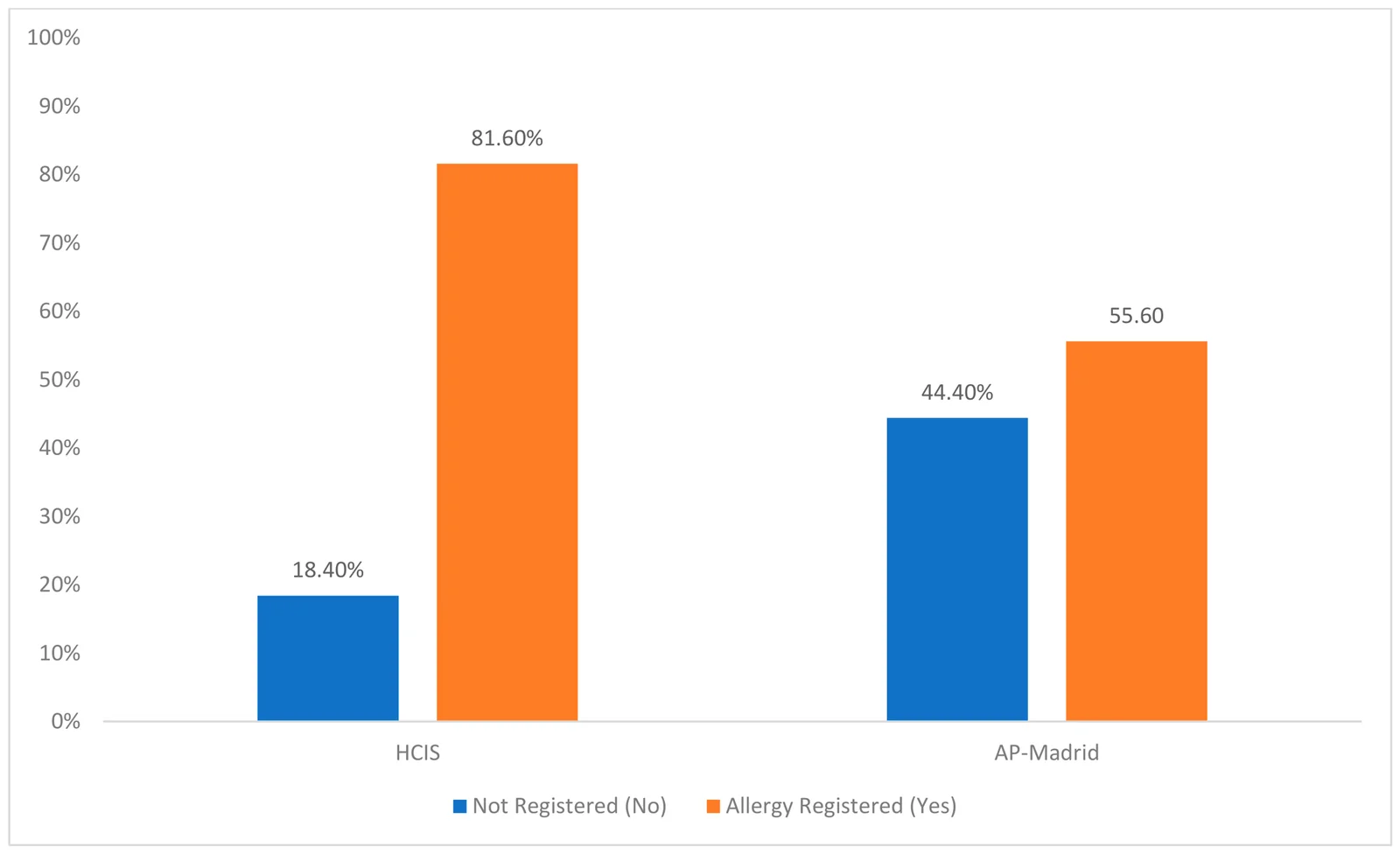
Comparison of the percentage of registered and unregistered cases in HCIS vs. AP-Madrid.

Agreement between the two clinical information systems was evaluated using Cohen’s kappa coefficient, yielding a value of −0.25 (95% CI: −0.39 to −0.12; *p* < 0.0001). This result indicates poor agreement (or slight disagreement), highlighting significant inconsistencies in how allergy data are synchronized or recorded in the two systems. Significant discrepancies were observed: 94 cases were documented exclusively in HCIS, while 36 cases were documented only in AP-Madrid. Simultaneous documentation in both systems occurred in only 88 cases; however, in 12 of these cases, the recorded medication allergies differed entirely between the two platforms. Finally, medication allergies were mentioned only in the free-text clinical narratives of five cases and were not documented in the structured allergy section of either system (Table 3). The proportion of cases with documented medication allergies was 81.6% in HCIS and 55.6% in AP-Madrid, while 18.4% and 44.4%, respectively, had no documented allergy record (Figure 1).

## 3. Discussion

The objective of this study was not to determine the superiority of one information system over another but rather to identify and characterize discrepancies between systems. The observed differences should therefore be interpreted as evidence of incomplete interoperability and inconsistency in allergy documentation across healthcare settings. A critical finding was the high proportion of allergy histories documented with incomplete data, particularly regarding reaction severity. In more than 90% of cases, severity was recorded as unknown, reflecting a profound deficiency in initial clinical characterization that substantially limited the utility of these records. These results are supported by Yang et al. [19], who recently demonstrated that medication allergy documentation in digital health records often lacks sufficient detail to be clinically actionable, particularly following adverse reactions.

Reaction severity is a fundamental element for therapeutic decision-making and the prevention of adverse events; therefore, its absence directly compromises patient safety. Our results align with the systematic review by Luri et al. [17], which observed considerable variation in how allergies are recorded across 17 different alert systems. This lack of standardization, specifically regarding data types and terminology, hinders the interoperability required to generate precise clinical alerts.

Although recommendations and contraindications were recorded for the majority of patients, a substantial proportion (nearly 30% in AP-Madrid) still lacked this information. This finding echoes the qualitative study by Fernando et al. [12], which identified barriers such as time constraints, system complexity, and a lack of awareness regarding the importance of documenting comprehensive clinical data. Furthermore, Radford et al. [20] demonstrated that implementing formalized history sheets significantly improved documentation, suggesting that our observed gaps could be mitigated through structured protocols.

Regarding the pharmacological groups involved, beta-lactams were the most frequently recorded. This is consistent with the findings of Rosado-Ingelmo et al. [21] at the Hospital Universitario Fundación Alcorcón, where NSAIDs and penicillins were the primary drivers of allergy alerts. The predominance of antibiotics, particularly beta-lactams, among medication allergy records may partly reflect their widespread use and the high frequency with which antibiotic allergy labels are reported in clinical practice. In addition, antibiotic allergy labels may originate from reactions that occurred many years earlier, for which information regarding the characteristics, severity, or timing of the original reaction is incomplete. Such labels may subsequently persist in electronic health records without formal allergy evaluation or reassessment, contributing to incomplete or poorly characterized documentation. The recently published ESCMID guidelines emphasize the importance of structured assessment of reported antibiotic allergies and appropriate evaluation of potentially inaccurate allergy labels [14]. These alerts frequently persist despite evidence that many patients labeled as allergic can tolerate these agents after appropriate evaluation, likely due to loss of sensitization over time and the limited cross-reactivity among different beta-lactam side chains [22,23]. In contrast, reactions associated with NSAIDs are more often attributable to non-allergic hypersensitivity mechanisms related to cyclooxygenase-1 inhibition rather than to true immunologically mediated allergy [23,24]. Additionally, the low representation of other groups in our study may reflect underreporting rather than lower incidence, especially given that 60.4% of records were classified as “poor” quality. Despite the overall low frequency of re-exposure observed in this cohort, the clinical significance of the identified positive re-exposure event should not be underestimated. Although two confirmed positive re-exposure events were identified, this illustrates the potentially serious consequences of incomplete or inconsistent medication allergy documentation. Alarmingly, a case of anaphylaxis was documented following an accidental re-exposure to amoxicillin in a patient with a history of hypersensitivity; this re-exposure resulted in anaphylactic shock due to the failure to document the allergy in the electronic health record. This incident underscores that even a single documentation error can lead to life-threatening consequences, transforming a preventable risk into a severe adverse event. Scientific evidence supports the finding that allergy history omissions are among the most dangerous discrepancies in electronic systems [25]. Studies in emergency settings have shown that incomplete documentation of severe reactions is directly linked to preventable iatrogenic anaphylaxis [26,27].

Apart from the reported case of anaphylaxis following accidental re-exposure, our study did not identify sufficient evidence to establish a direct relationship between documentation discrepancies and additional adverse patient outcomes. Given the retrospective design and reliance on routinely documented clinical records, it was not possible to determine whether all discrepancies resulted in medication errors or adverse drug reactions. Nevertheless, the high frequency of documentation inconsistencies represents an important patient safety concern, as inaccurate or incomplete allergy records may contribute to inappropriate prescribing, unnecessary avoidance of first-line therapies, or preventable re-exposure to allergenic medications.

Importantly, our study identified five cases in which a medication allergy was documented exclusively within free-text clinical notes and absent from the structured allergy modules of both AP-Madrid and HCIS. Although no adverse outcomes were documented in these patients, such omissions represent a significant safety concern because allergies recorded only in unstructured text are not incorporated into electronic allergy lists and therefore may not activate clinical decision support alerts. Consequently, healthcare professionals may remain unaware of these allergies unless they manually review previous clinical notes, increasing the risk of inadvertent prescribing of contraindicated medications.

Finally, the agreement analysis between HCIS and AP-Madrid revealed poor agreement, reflected by a negative Cohen’s kappa coefficient (κ = −0.25). This finding indicates that the level of agreement between the two systems was poorer than would be expected by chance alone, reflecting substantial inconsistencies in medication allergy documentation rather than a statistical anomaly. The negative kappa coefficient is likely explained by the large number of discordant records documented exclusively in one of the two systems, together with the asymmetric distribution of allergy documentation between HCIS and AP-Madrid. However, agreement statistics such as Cohen’s kappa should be interpreted cautiously as they may be influenced by the prevalence and distribution of observations across categories. Therefore, these findings should be considered together with the broader clinical context and the descriptive results of the study.

Notably, the hospital system contained nearly three times the number of exclusive records (*n* = 94) compared with primary care (*n* = 36). This asymmetric profile suggests that the hospital environment serves as the primary setting for identifying and documenting new reactions to medications commonly used in acute care, such as penicillins, cephalosporins, and nonsteroidal anti-inflammatory drugs (NSAIDs). In contrast, primary care records may not be updated consistently following hospital encounters because the two clinical information systems are not automatically synchronized and rely on independent documentation workflows. Furthermore, differences in clinical documentation practices between healthcare settings, variations in the timing and frequency of record updates, incomplete transfer of clinically relevant information between systems, and the recording of allergy information exclusively in free-text clinical notes rather than structured allergy modules may all contribute to the poor agreement observed.

From a clinical perspective, these discrepancies are particularly concerning because incomplete or discordant medication allergy documentation may increase the risk of inappropriate prescribing, unnecessary avoidance of first-line therapies, duplicate or inconsistent allergy records, and failure to recognize clinically relevant allergies during subsequent episodes of care. The failure to transfer updated allergy information to the AP-Madrid registry effectively perpetuates a patient safety risk following hospital discharge and compromises the reliability of electronic health records during clinical decision-making. These findings underscore the importance of ensuring that medication allergy information is consistently available across healthcare settings to support safe prescribing and continuity of care. Future interoperability initiatives may benefit from standardized approaches for the structured exchange of allergy and intolerance information across healthcare systems. Modern interoperability frameworks, such as standardized electronic health data exchange models, may facilitate more consistent allergy documentation, improve synchronization between care settings, and ultimately enhance patient safety during transitions of care [28]. To understand the root causes of these inconsistencies, it is necessary to examine the broader clinical environment. The observed discrepancies are likely multifactorial and may reflect a combination of technical, organizational, and human factors within the healthcare system. From a technical perspective, incomplete interoperability between clinical information systems may limit synchronization of allergy records across care settings. Organizationally, duplicate documentation workflows and time constraints may discourage consistent updating of allergy information in multiple platforms. In addition, variability in clinician documentation practices, differences in allergy reconciliation processes, and limited awareness regarding the clinical implications of incomplete allergy records may further contribute to inconsistencies.

Our findings are consistent with studies conducted in other healthcare systems, which have similarly reported substantial discrepancies in medication allergy documentation across electronic health records. However, the magnitude of disagreement observed in our study appears to be greater than that reported by Rukasin et al. (2020), who found that 15% of patients had discrepancies between information systems, often due to the absence of automated reconciliation tools [18]. Likewise, Novotny et al. (2025) described penicillin allergy labels as “islands of information” that remain isolated within different electronic health records because of limited interoperability [29]. Similarly, Ramsey and Staicu [30] highlighted that incomplete or inaccurate allergy documentation contributes to the persistence of unverified allergy labels, unnecessary avoidance of first-line therapies, and increased healthcare costs. From a practical perspective, these findings highlight the need to improve interoperability between AP-Madrid and HCIS by enabling structured medication allergy records to be automatically synchronized across healthcare settings, ensuring that clinicians have access to complete and up-to-date allergy information regardless of the point of care. Improved interoperability would strengthen clinical decision support systems by enhancing the accuracy of allergy alerts, facilitating safer prescribing during transitions of care, and reducing duplicate documentation and preventable medication errors. However, interoperability alone is unlikely to fully address the deficiencies identified in this study. Healthcare institutions should also implement standardized documentation protocols, require the recording of essential allergy information (including the suspected medication, reaction characteristics, severity, and documentation date), integrate clinical decision support prompts that alert clinicians when mandatory documentation fields are incomplete, and promote regular education, periodic audits, and feedback to improve documentation practices. Collectively, these measures would improve the quality, completeness, consistency, and reliability of medication allergy documentation, strengthen continuity of care, optimize allergy management across healthcare settings, and ultimately enhance patient safety. Based on these findings, the main medication allergy documentation problems identified in this study, their potential clinical implications, and the corresponding proposed interventions are summarized in Table 4.

Although the present study was conducted within a single tertiary hospital and its associated primary care network, the challenges identified regarding medication allergy documentation, incomplete interoperability, and discrepancies between primary care and hospital information systems have also been reported in other healthcare settings. Therefore, while the magnitude of the observed discrepancies may vary depending on local electronic health record infrastructure, clinical workflows, and documentation practices, the findings are likely to be relevant to healthcare organizations facing similar challenges in the management of electronic medication allergy records.

### Study Limitations

Study Design and Bias: First, its retrospective design inherently relies on the accuracy and completeness of existing electronic health records (EHRs), introducing potential information bias related to the quality of historical documentation. Because data were collected after clinical care had already occurred, the investigators had no control over how allergy information was originally documented, resulting in missing, incomplete, or inconsistently recorded variables. Furthermore, the retrospective nature of the study precluded standardized data collection and did not allow clarification or verification of ambiguous documentation with the treating clinicians or patients. Additionally, patients were initially identified through the hospital records and subsequently matched with the primary care system, a process that may have inherently influenced the observed discrepancies. Furthermore, the inclusion of only patients with available records in both information systems may have introduced selection bias and may limit the generalizability of the findings to patients receiving care outside these settings.

Completeness and Misclassification: The high proportion of missing data, particularly regarding reaction severity (which was frequently recorded as “unknown”), limited our ability to fully characterize clinical phenotypes and the true clinical impact of the recorded allergies. Furthermore, because the analysis was based exclusively on routinely documented electronic health records without supplementary patient interviews or clinical verification, it was not possible to definitively distinguish between true immunologically mediated drug allergies, drug intolerances, predictable adverse drug reactions, or “administrative” allergy labels that may no longer have been clinically relevant. Consequently, some degree of misclassification may have occurred. However, this limitation reflects routine clinical practice, where these entities are frequently documented interchangeably within electronic health records, and may itself have contributed to the observed discrepancies and poor documentation quality between the two information systems.

Subjectivity in Assessment: Although data extraction was performed using predefined criteria and subsequently validated by a senior researcher, some degree of subjectivity in the assessment of clinical documentation cannot be completely excluded.

Sensitivity Analysis: No formal sensitivity analyses were performed to evaluate the robustness of the findings under alternative analytical assumptions. However, all eligible patients meeting the predefined inclusion criteria during the study period were included in the analyses, thereby reducing the potential impact of selection-related variability. Nevertheless, future studies could incorporate sensitivity analyses to further assess the stability and robustness of the observed findings.

Potential confounding factors, such as patient age, comorbidities, frequency of healthcare utilization, or clinical complexity, were not specifically evaluated because the primary objective of this study was to descriptively assess medication allergy documentation and agreement between two clinical information systems rather than to investigate causal associations. Nevertheless, these factors may influence documentation practices and should be considered in future studies employing analytical designs.

Scope and Outcomes: The present study did not directly assess downstream clinical outcomes, such as prescribing substitutions, healthcare utilization, or financial costs. Therefore, these consequences should be interpreted as potential implications supported by the existing literature rather than direct observations from this dataset.

Generalizability: Finally, as this study was conducted within a single tertiary hospital and its associated primary care network in the Community of Madrid, the findings are specific to the local digital infrastructure (HCIS and AP-Madrid). Consequently, the observed discrepancy rates and technical interoperability challenges may not be fully generalizable to other healthcare regions utilizing different digital ecosystems or integration protocols. Although similar challenges related to medication allergy documentation and interoperability have been reported in other healthcare settings, the exact magnitude of the observed discrepancies may differ according to local electronic health record infrastructure, clinical workflows, and documentation practices. Therefore, caution should be exercised when extrapolating the quantitative findings of this study to other healthcare systems.

## 4. Materials and Methods

### 4.1. Study Design and Population

This retrospective cohort study analyzed a shared patient population documented in both the primary care information system (AP-Madrid) and the Hospital Clinical Information System (HCIS) at La Paz University Hospital. A retrospective cohort design was selected because the study aimed to evaluate the consistency of medication allergy documentation between two existing electronic clinical information systems using routinely collected real-world data. This design enabled the assessment of documentation practices and inter-system agreement over a defined study period without influencing clinical practice or documentation behavior. Furthermore, it allowed the identification of documentation discrepancies and medication re-exposure events under routine healthcare conditions. AP-Madrid and HCIS are distinct electronic clinical information systems used in primary care and hospital settings, respectively, with no automatic synchronization between them. Both systems include dedicated structured sections for medication allergy documentation; however, documentation workflows and updating processes may differ between care settings:

- In AP-Madrid (Primary Care Setting): General practitioners record the allergy and its severity using standardized codes (e.g., ICD) or free text. Symptoms are described via free-text fields, while the implicated medication can be selected from a predefined list of active ingredients authorized in primary care or entered as free text. The dates of entry, removal, or modification are automatically logged by the system. Crucially, the electronic prescribing module does not feature any active alert mechanisms or pop-up warnings for the registered drug, meaning that the prescribing workflow remains uninterrupted and relies solely on the clinician manually reviewing the documented allergy history.
- In HCIS (Hospital Setting): Medical and nursing staff can record or modify allergy data during a patient’s admission. The allergy and its severity are entered using codes (ICD) or free text, and both symptom descriptions and clinical evaluation status are documented via free-text notes. Medications are registered by active ingredient using an institutional dropdown list or as free text; additionally, the system allows selection of entire therapeutic subgroups, such as antibiotics and non-steroidal anti-inflammatory drugs (NSAIDs). This entry does not require validation by the Allergology or Clinical Pharmacology departments. The dates of entry or removal are automatically recorded. When a medication allergy is registered within this structured module, the hospital prescribing system triggers an overridable alert for the drug or therapeutic subgroup, displaying a warning notice that requires explicit confirmation (“Do you want to continue? Yes/No”) to proceed with the prescription.

The study protocol was reviewed and approved by the La Paz University Hospital Ethics Committee (PI-5775, 29 June 2023). Given the retrospective design and adherence to institutional and national legislation, the requirement for written informed consent was waived. Each case was assigned a unique, pseudonymized identification number within the Case Report Form (CRF) to ensure patient confidentiality and prevent direct identification.

The study population included patients with documented clinical records in both the Hospital Clinical Information System (HCIS) and the primary care system (AP-Madrid). For the purpose of this study, medication allergy documentation was defined as information recorded within the dedicated structured allergy sections of AP-Madrid and HCIS. Mentions identified exclusively within free-text clinical narratives were analyzed separately and were not considered structured allergy documentation. The term “documentation” therefore refers to the presence and recording of medication allergy information within these structured sections of each clinical information system.

Eligible patients were identified through the HCIS “Lists and Statistics” module by systematically searching for documented “allergy” diagnoses across all hospital departments and emergency care settings between 1 January and 31 December 2022. To ensure comprehensive data capture, clinical follow-up for suspected drug allergy evaluations was extended through 31 May 2023. This allowed the inclusion of all relevant diagnostic outcomes and subsequent updates to the patients’ records. To maintain data integrity, duplicate records were eliminated, and each patient was treated as a single case, even if evaluated by multiple specialties for the same clinical process.

**Figure 2:**
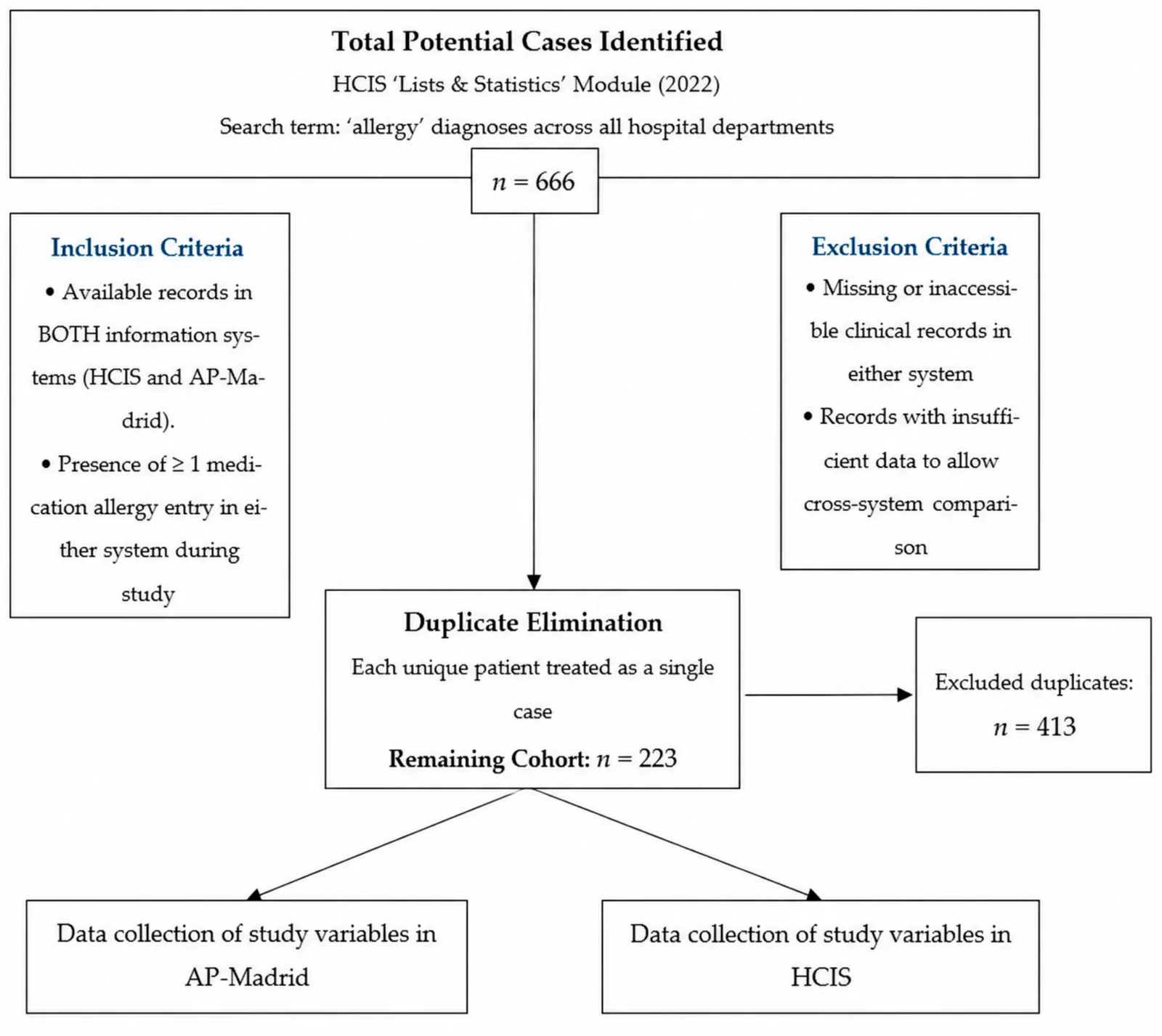
Flowchart of patient selection and study enrolment.

Inclusion criteria were: (i) patients with available records in both systems and (ii) patients with at least one medication allergy entry in either system during the study period. Conversely, exclusion criteria included: (i) patients without accessible records in one of the systems and (ii) records with insufficient information to allow comparison between systems. All eligible patients identified during the study period who met the predefined inclusion criteria were included, thereby minimizing investigator-related selection bias (Figure 2).

Data linkage between the two independent clinical information systems was performed deterministically using each patient’s unique Regional Personal Health Identification Number (Código de Identificación Personal Autonómico, CIPA). As this approach included only patients with records available in both information systems, some degree of selection bias cannot be excluded, particularly because patients without documentation in one system or cross-system records were not eligible for inclusion. Consequently, the findings may not be fully representative of all patients with documented medication allergies.

Data extraction and classification of medication allergy documentation were performed by one researcher using predefined criteria. All identified patient records were manually reviewed after retrieval through the HCIS keyword search. In addition to the structured allergy modules, the corresponding free-text clinical documentation was examined to identify medication allergies that had not been recorded as structured allergy entries. Free-text documentation was also reviewed to obtain additional clinical information, particularly descriptions of allergic symptoms, when these data were absent from the structured allergy record. Information identified exclusively in free-text fields was used to characterize documentation discrepancies and supplement the extraction of clinical variables. However, only structured allergy registrations were included in the comparison between HCIS and AP-Madrid and the assessment of documentation quality. All records retrieved through the initial keyword search were manually reviewed to confirm eligibility. Records referring exclusively to non-medication allergies (e.g., food, pollen, animal dander, or other environmental allergens) were excluded according to the predefined inclusion criteria. No data imputation was performed during the study. When specific documentation elements, such as reaction severity or symptom descriptions, remained unavailable after review of both the structured allergy records and the corresponding free-text clinical documentation, these variables were considered missing and contributed to the assessment of documentation quality. To enhance data accuracy and consistency, all extracted data were subsequently reviewed and validated by a senior researcher. All study variables, including the parameters used to assess documentation quality, were defined a priori to minimize subjectivity. Because the initial data extraction was performed by a single reviewer and the second reviewer was involved in data validation rather than independent duplicate extraction, formal inter-rater reliability statistics were not calculated. Any uncertainties identified during the validation process were resolved through discussion and consensus. When discrepancies between AP-Madrid and HCIS were identified, no attempt was made to reconcile or adjudicate conflicting information, as the objective of this study was to evaluate the existence, frequency, and characteristics of these discrepancies under routine clinical conditions. Therefore, each system was analyzed independently according to its recorded documentation.

### 4.2. Sample Size Calculation

To evaluate the agreement between both electronic health record systems using Cohen’s kappa coefficient, an a priori sample size estimation was performed. To establish a highly conservative and rigorous estimation, the parameters were defined as follows: a 5% significance level (95% confidence), 80% statistical power, and target minimum expected kappa coefficient of 0.20, which was selected as the standard baseline threshold for detecting slight agreement beyond chance (according to Landis and Koch benchmarks) and establishing a conservative lower bound for non-synchronized systems. Additionally, maximum potential variance was assumed by setting the marginal rating probabilities symmetrically at 50%.

Under these criteria, the minimum required sample size was calculated to be 197 unique patients. Since the final study cohort comprised 223 patients, this study provided adequate statistical power to identify and analyze documentation discrepancies and cross-system agreement regarding medication allergies between primary care and the hospital environment.

### 4.3. Study Variables and Quality Assessment

The study variables were recorded in a CRF and included sociodemographic and clinical variables. Patient age, sex, comorbidities, and personal histories of allergies and adverse drug reactions were collected from both the primary care (AP-Madrid) and the hospital (HCIS) information systems. For each medication allergy diagnosis, we recorded the date of diagnosis, the specific diagnosis (including the documented clinical manifestation associated with the reported medication allergy, such as urticaria, rash, angioedema, anaphylaxis, or other documented hypersensitivity reactions), reaction severity, clinical outcome, and suspected drugs and/or therapeutic groups involved, as well as associated clinical recommendations and contraindications. In addition, specialized evaluations were documented, including evaluation dates, the types of tests performed (diagnostic algorithms, skin or patch tests, in vitro tests, drug provocation tests, and sensitization studies), the diagnosis derived from the evaluation, and the drugs or therapeutic groups ultimately considered responsible. Regarding the administrative record of the allergy, we collected documentation and removal dates and recorded severity and informational notes. Subsequent pharmacological prescriptions were documented, with their respective start and end dates and administered doses. Finally, any adverse drug reactions occurring after re-exposure (both positive and negative) were documented, including diagnosis, causality assessment, severity, and clinical outcome.

Finally, medication re-exposure was operationally defined and comprehensively tracked across three distinct levels of the clinical workflow: medical ordering (order entry), computerized inpatient nursing administration charts within HCIS, and community pharmacy dispensing logs via AP-Madrid (outpatient dispensing). To ensure robust tracking of these events, clinical and pharmacotherapeutic follow-up was strictly extended for an additional five months post-index (through 31 May 2023). For every identified re-exposure event, an in-depth manual electronic health record (EHR) chart review was performed by the researchers to thoroughly reconstruct the clinical timeline, verify the administrative status of the allergy module at the time of prescription, and cross-reference clinical progress notes. Any adverse drug reactions occurring after re-exposure (both positive and negative) during this period were thoroughly documented, including diagnosis, causality assessment, severity, and clinical outcome.

The quality of medication allergy documentation was compared between the two systems and evaluated for completeness. To standardize this assessment, four key documentation parameters were evaluated: (1) allergy documentation, (2) documentation date, (3) reaction severity, and (4) notes describing symptoms. These parameters were selected based on previous studies evaluating the quality of medication allergy documentation and their recognized clinical relevance, as they represent essential elements for the interpretation and safe management of medication allergy records. Similar to previous studies, they were used as practical indicators of documentation quality rather than as a formally validated documentation quality scoring system [31]. A symptom description was considered “present” only when it was complete, providing sufficient information on symptom duration, severity, and the clinical management applied for resolution.

Documentation quality was classified according to the completeness of the recorded information based on the number of predefined documentation parameters present in each record. This pragmatic classification was developed to provide a standardized and reproducible method for comparing documentation quality between the two clinical information systems. Records containing all four parameters were considered to have the highest level of documentation quality, whereas records with fewer documented parameters were considered progressively less complete. Accordingly, documentation was classified as follows:

- Adequate: All four parameters were present.
- Moderate: Three parameters were included.
- Poor: Two or fewer parameters were recorded.

Pharmacological agents implicated in medication allergies were standardized and classified according to the Anatomical Therapeutic Chemical (ATC) classification system hierarchy. For combination products containing more than one active substance, mapping was handled by analyzing each active component individually under its respective ATC code to ensure precise frequency tracking or, when appropriate, under the specific combination ATC code to maintain data integrity.

### 4.4. Statistical Analysis Description

Categorical variables were summarized as frequencies and percentages, while continuous variables were described using means and standard deviations or medians and interquartile ranges, depending on their distribution.

Agreement between AP-Madrid and HCIS regarding the presence of medication allergy documentation, defined as the primary endpoint, was assessed using Cohen’s kappa coefficient along with its asymptotic 95% confidence interval (95% CI).

In contrast, the quality of medication allergy documentation was analyzed descriptively according to predefined categories (adequate, moderate, and poor). The distribution of implicated drugs and therapeutic groups was also explored descriptively. Statistical analyses were performed using R software (R Core Team, 2019; Vienna, Austria), available at https://www.R-project.org/ (accessed on 8 July 2026). For all hypothesis testing, a *p*-value < 0.05 was considered statistically significant.

## 5. Conclusions

This study revealed critical discrepancies in medication allergy documentation across healthcare settings, characterized by poor documentation quality and limited interoperability between primary care and hospital information systems. These deficiencies may compromise patient safety by contributing to the unnecessary avoidance of first-line therapies, increasing the risk of preventable medication-related adverse events, and increasing healthcare costs. Future improvements may require not only standardized documentation protocols but also structured interoperability strategies, clearer allergy reconciliation procedures, and greater integration of medication allergy documentation into routine clinical workflows. Collectively, these measures have the potential to improve documentation quality, strengthen continuity of care, and enhance patient safety across healthcare settings.

## Figures and Tables

**Table 1:** Population characteristics and allergy history in HCIS.

Variable	Category	Overall
Sample size	*n*	223
Age (mean (SD))	Mean (SD)	64.66 (26.30)
Sex (%)	Female	128 (57.4%)
	Male	95 (42.6%)
Personal history of allergies	No	177 (79.4%)
	Yes	46 (20.6%)
Allergy Evaluation and Documentation		
Severity (Evaluation)	Unknown	203 (91.0%)
	Mild	3 (1.3%)
	Severe	17 (7.6%)
Recommendations recorded	Recorded	217 (97.3%)
	Not recorded	6 (2.7%)
Departmental Distribution and Medication Groups		
Variable	Detail	*n* (%)
Department	Allergology	71 (31.8%)
	Not studied	109 (48.9%)
	Others	41 (18.4%)
	External: Hospital Rey Juan Carlos	1 (0.4%)
	External: Ramón y Cajal Hospital	1 (0.4%)
	Antibiotics	105 (50.2%)
	Analgesics/Pyrazolones	37 (17.7%)
	Antineoplastic agents	6 (2.9%)
	Heparins	5 (2.4%)
	Contrast media	3 (1.4%)
	ACE inhibitors	3 (1.4%)
	Antiepileptics	3 (1.4%)
	Others	47 (22.5%)
Re-exposure and documentation quality		
Variable	Outcome	*n* (%)
Re-exposure	No	217 (98.2%)
	Positive	2 (0.9%)
	Negative	2 (0.9%)
Confirmed diagnosis	No	219 (99.1%)
	Yes	2 (0.9%)
Causality	No	219 (99.1%)
	Yes	2 (0.9%)
Severity (re-exposure)	Unknown	219 (99.1%)
	Mild	1 (0.5%)
	Severe	1 (0.5%)
Documentation quality	Poor	134 (60.4%)
	Moderate	71 (32.0%)
	Adequate	17 (7.7%)

**Table 2:** Population characteristics and allergy history in AP-Madrid.

Variable	Category	Results
Allergy Evaluation and Documentation		
Personal history of allergies recorded	No	113 (50.7%)
	Yes	110 (49.3%)
Severity (evaluation) Recommendations recorded	Unknown	202 (90.6%)
	Mild	3 (1.3%)
	Moderate	1 (0.5%)
	Severe	17 (7.6%)
	Recorded	146 (65.5%)
	Not recorded	77 (34.5%)
Re-exposure and Documentation Quality		
Medication group (allergy evaluation)	Antibiotics	82 (36.44%)
	NSAIDs	46 (20.44%)
	Analgesics (pyrazolones + anilides)	35 (15.56%)
	Opioids	15 (6.67%)
	Antispasmodics	8 (3.56%)
	Cardiovascular drugs	11 (4.89%)
	Local anesthetics	3 (1.33%)
	Antineoplastic agents	2 (0.89%)
	Others	23 (10.22%)
Re-exposure	No	183 (82.1%)
	Positive	1 (0.5%)
	Unknown	39 (17.4%)
Confirmed diagnosis	No	223 (100.0%)
Causality	No	223 (100.0%)
Severity (re-exposure)	Unknown	223 (100.0%)
Documentation quality	Poor	138 (61.9%)
	Moderate	76 (34.1%)
	Adequate	9 (4%)

**Table 3:** Agreement between HCIS and AP-Madrid allergy records.

	AP-Madrid (Registered)	AP-Madrid (Not Registered)	Total HCIS
HCIS (Registered)	88	94	182
HCIS (Not Registered)	36	5	41
Total AP-Madrid	124	99	223

Note: Cohen’s kappa coefficient κ = −0.25 (95% CI: −0.39 to −0.12; *p* < 0.0001). Negative values or values near zero suggest that the observed agreement is lower than what would be expected by chance alone. Out of the 88 overlapping cases documented in both platforms, 12 presented discordant medication allergy labels between systems.

**Table 4:** Medication allergy documentation problems, clinical impacts, and proposed interventions.

Problem Identified	Potential Clinical Impact	Proposed Intervention
Incomplete and poor-quality allergy documentation	Limits clinical interpretation and safe therapeutic decision-making.	Standardize documentation of essential allergy information.
Reaction severity frequently recorded as unknown (>90%)	Limits assessment of the clinical significance of reported reactions.	Require structured severity recording and prompts for missing information.
Poor agreement between AP-Madrid and HCIS (κ = −0.25)	Clinicians may have inconsistent allergy information across care settings.	Improve interoperability and synchronization between systems.
Allergy records documented exclusively in one system (HCIS: *n* = 94; AP-Madrid: *n* = 36)	Relevant allergy information may be unavailable in the other care setting.	Implement automatic bidirectional synchronization of allergy records.
Allergies documented only in free text (*n* = 5)	Allergies may be overlooked and may not trigger electronic alerts.	Transfer relevant free-text allergy information to structured allergy modules.
Accidental re-exposure to amoxicillin due to undocumented allergy	May result in preventable severe adverse reactions, including anaphylaxis.	Ensure prompt documentation and cross-system updating of confirmed allergies
Differences in documentation and updating practices between settings	May result in incomplete, inconsistent, or outdated allergy information.	Standardize documentation and updating procedures across care settings.

## Data Availability

The raw data supporting the conclusion of this article will be made available by the authors upon reasonable request, without undue reservation.

## Supplementary Materials

The following supporting information can be downloaded at https://www.mdpi.com/article/10.3390/ph19081259/s1. Supplementary Table S1: Pharmacological distribution of medication allergy records in the Hospital Clinical Information System (HCIS); Supplementary Table S2: Pharmacological distribution of medication allergy records in the primary care information system (AP-Madrid).

## Abbreviations

The following abbreviations are used in this manuscript:

Abbreviation	Full Term
ADR	Adverse Drug Reaction
AP-Madrid	Primary Care Records System of the Community of Madrid
CRF	Case Report Form
HCIS	Hospital Clinical Information System
IdiPAZ	La Paz University Hospital Health Research Institute
NSAIDs	Nonsteroidal Anti-inflammatory Drugs
UAM	Universidad Autónoma de Madrid
WHO	World Health Organization
