# Supplementary material for: Discrepancies and Quality of Medication Allergy Documentation Between Primary Care and Hospital Information Systems: A Retrospective Cohort Study
Source: Pharmaceuticals (Basel). 2026 Aug 10;19(8):1259. doi: 10.3390/ph19081259 (PMC13516464; doi:10.3390/ph19081259)
Supplement: Supplementary file 1 [file pharmaceuticals-19-01259-s001.zip › pharmaceuticals-4457370-supplementary.pdf]

Supplementary Material

Table S1 - Registered Medications HCIS

Medication	Count	Percentage	ATC Code	Medication
Metamizol	34	16.27	N02BB02	Analgesics
Amoxicilina	11	5.26	J01CA04	Penicillins
Bencilpenicilina	11	5.26	J01CE01	Penicillins
Penicilinas	11	5.26	J01C	Therapeutic group
Amoxicilina - clavulanico (incluye augmentine)	8	3.83	J01CR02	Combinations of penicillins. beta-lactamase inhibitors
Ceftriaxona	7	3.35	J01DD04	Cephalosporins
Vancomicina	7	3.35	J01XA01	Glycopeptide antibacterials
Cefepima	6	2.87	J01DE01	Cephalosporins
Eritromicina	5	2.39	J01FA01	Macrolides
Estreptomicina	5	2.39	J01GA01	Aminoglycosides
Procaina	4	1.91	N01BA02	Local anesthetics
Neomicina	3	1.44	J01GB05	Aminoglycosides
Cloxacilina	3	1.44	J01CF02	Penicillins
Enalaprilo	2	0.96	C09AA02	ACE inhibitors
Codeina	2	0.96	R05DA04	Antitussives
Dexketoprofeno	2	0.96	M02AA27	Topical NSAIDs
Ciprofloxacino	2	0.96	J01MA02	Quinolones
Tramadol	2	0.96	N02AX02	Analgesics
Leflunomida	2	0.96	L04AA13	Immunosuppressant s
Alopurinol	2	0.96	M04AA01	Anti-gout agents
Sulfamidas	2	0.96	J01E	Therapeutic group
Ibuprofeno	2	0.96	M01AE01	NSAIDs
Metronidazol	2	0.96	J01XD01	Antibacterials Imidazole derivatives
Bemiparina	2	0.96	B01AB12	Heparins

Cefotaxima	2	0.96	J01DD01	Cephalosporins
Quinolonas	2	0.96	J01M	Therapeutic group
Sulfametoxazol/trimetoprim	2	0.96	J01EE01	Combinations
Levofloxacino	2	0.96	J01MA12	Quinolones
Linezolid	2	0.96	J01XX08	Other antibiotics
Carboplatino	2	0.96	L01XA02	Antineoplastics
Amlodipina	2	0.96	C08CA01	Calcium channel blockers
Tetraciclinas	2	0.96	J01A	Therapeutic group
Butilescopolamina	2	0.96	A03BB04	Antispasmodics
Fentanilo	1	0.48	N02AB03	Opioid analgesics
Movicol	1	0.48	A06AD65	Laxatives
Captoprilo	1	0.48	C09AA01	ACE inhibitors
Ertapenem	1	0.48	J01DH03	Carbapenems
Rocuronio bromuro	1	0.48	M03AC09	Muscle relaxants
Visipaque (iodixanol)	1	0.48	V08AB08	Contrast media
Paracetamol	1	0.48	N02BE01	Analgesics
Piperacilina + tazobactam	1	0.48	J01CR05	Combinations
Latex	1	0.48	-	Not applicable
Sulfadiazina	1	0.48	J01EC02	Sulfonamides
Oxitetraciclina	1	0.48	J01AA06	Tetracyclines
Teicoplanina	1	0.48	J01XA02	Other antibiotics
Vincristina	1	0.48	L01CA02	Antineoplastics
Plaquetas	1	0.48	B05AX01	Blood derivatives
Ranitidina	1	0.48	A02BA02	H2 antihistamines
Carboplatino-paclitaxel	1	0.48	L01XA02/L01CD01	Antineoplastics
Hioscina	1	0.48	A03BB01	Antispasmodics
Mitomicina	1	0.48	L01DC03	Antineoplastics
Metoclopramida	1	0.48	A03FA01	Antiemetics
Enoxaparina	1	0.48	B01AB05	Heparins
Cromo	1	0.48	A12CB02	Trace elements

Haloperidol	1	0.48	N05AD01	Antipsychotics
Pantoprazol	1	0.48	A02BC02	Proton pump inhibitors
Acetilcisteina	1	0.48	R05CB01	Mucolytics
Dexametasona	1	0.48	H02AB02	Corticosteroids
Ceftazidima + avibactam	1	0.48	J01DI56	Combinations
Carbapenems	1	0.48	J01DH	Therapeutic group
Corticoides	1	0.48	H02	Therapeutic group
Fenitoina	1	0.48	N03AB02	Antiepileptics
Bromhexina	1	0.48	R05CB02	Mucolytics
Carbamazepina	1	0.48	N03AF01	Antiepileptics
Rifaximina	1	0.48	A07AA11	Intestinal anti-infectives
Clortalidona	1	0.48	C03BA04	Diuretics
Nadroparina	1	0.48	B01AB06	Heparins
Heparina sodica	1	0.48	B01AB01	Heparins
Iopamiro	1	0.48	V08AB05	Contrast media
Bisoprolol	1	0.48	C07AB07	Beta-blockers
Ampicilina	1	0.48	J01CA01	Penicillins
Amiodarona	1	0.48	C01BD01	Antiarrhythmics
Benznidazol	1	0.48	P01CA02	Antiprotozoals
Povidona iodada (betadine)	1	0.48	D08AG02	Antiseptics
Pirimetamina	1	0.48	P01BD01	Antiprotozoals
Meropenem	1	0.48	J01DH02	Carbapenems
Mepolizumab	1	0.48	R03DX09	Respiratory agents
Ropinirol	1	0.48	N04BC04	Antiparkinson agents
Apomorfina	1	0.48	N04BC07	Antiparkinson agents
Fluconazol	1	0.48	J02AC01	Antifungals
Cicloplejico	1	0.48	S01FA04	Mydriatics
Rituximab	1	0.48	L01XC02	Antineoplastics
Levetiracetam	1	0.48	N03AX14	Antiepileptics

Amitriptilina	1	0.48	N06AA09	Antidepressants
Hierro (iii) (carboximaltosa)	1	0.48	B03AB05	Iron preparations
Hierro (iii) sacarosa	1	0.48	B03AB02	Iron preparations
Iomeron	1	0.48	V08AB11	Contrast media

Table S2 - Registered Medications AP-Madrid

R jinhfynts	Htzsy	Ujwhjsyflj	FYH Htij	FYH Lwtzu
**Amoxicilina**	35	15.56	J01CA04	Penicillins w/ extended spectrum
**Metamizol**	31	13.78	N02BB02	Pyrazolones
**Ibuprofeno**	18	8.00	M01AE01	Propionic acid derivatives
**Ácido Acetilsalicílico**	14	6.22	N02BA01	Salicylic acid and derivatives
**Tramadol**	12	5.33	N02AX02	Opioids
**Ampicilina**	10	4.44	J01CA01	Penicillins w/ extended spectrum
**Naproxeno**	8	3.56	M01AE02	Propionic acid derivatives
**Levofloxacino**	7	3.11	J01MA12	Fluoroquinolones
**Ceftriaxona**	7	3.11	J01DD04	3rd generation cephalosporins
**Estreptomicina**	6	2.67	J01GA01	Aminoglycosides
**Hioscina (Butylbrombromide)**	6	2.67	A03BB01	Belladonna semi-synthetic
**Diclofenaco**	6	2.67	M01AB05	Acetic acid derivatives

**Amlodipino**	5	2.22	C08CA01	Dihydropyridine derivatives
**Cefixima**	5	2.22	J01DD08	3rd generation cephalosporins
**Ciprofloxacino**	4	1.78	J01MA02	Fluoroquinolones
**Vancomicina**	4	1.78	J01XA01	Glycopeptide antibacterials
**Paracetamol**	4	1.78	N02BE01	Anilides
**Codeína**	3	1.33	R05DA04	Opium alkaloids
**Metronidazol**	3	1.33	J01XD01	Imidazole derivatives
**Hidroclorotiazida**	3	1.33	C03AA03	Low-ceiling diuretics. thiazides
**Lidocaína**	2	0.89	N01BB02	Amides
**Enalapril**	2	0.89	C09AA02	ACE inhibitors
**Meropenem**	2	0.89	J01DH02	Carbapenems
**Cloranfenicol**	2	0.89	J01BA01	Amphenicols
**Metoclopramida**	2	0.89	A03FA01	Propulsives
**Tapentadol**	2	0.89	N02AX06	Opioids
**Procaína**	1	0.44	N01BA02	Esters of aminobenzoic acid
**Captopril**	1	0.44	C09AA01	ACE inhibitors. plain
**Eritromicina**	1	0.44	J01FA01	Macrolides
**Azitromicina**	1	0.44	J01FA10	Macrolides

**Piperacilina**	1	0.44	J01CA12	Penicillins w/ extended spectrum
**Tazobactam**	1	0.44	J01CR05	Penicillins + inhibitors
**Ticlopidina**	1	0.44	B01AC05	Platelet aggregation inhibitors
**Alopurinol**	1	0.44	M04AA01	Preparations inhibiting uric acid
**Ambroxol**	1	0.44	R05CB06	Mucolytics
**Bromazepam**	1	0.44	N05BA08	Benzodiazepine derivatives
**Ranitidina**	1	0.44	A02BA02	H2-receptor antagonists
**Paclitaxel**	1	0.44	L01CD01	Taxanes
**Carboplatino**	1	0.44	L01XA02	Platinum compounds
**Clotrimazol**	1	0.44	D01AC01	Imidazole and triazole derivatives
**Linezolid**	1	0.44	J01XX08	Antibacterials for systemic use
**Aztreonam**	1	0.44	J01DF01	Monobactams
**Fentanilo**	1	0.44	N02AB03	Phenylpiperidine derivatives
**Carbamazepina**	1	0.44	N03AF01	Carboxamide derivatives
**Rifaximina**	1	0.44	A07AA11	Antibiotics
**Pregabalina**	1	0.44	N03AX16	Antiepileptics
**Colistina**	1	0.44	J01XB01	Polymyxins
**Fosfomicina**	1	0.44	J01XX01	Antibacterials for systemic use
